# Supplementary material for: Wavefront Shaping of Scattering Forces Enhances Optical Trapping of Levitated Nanoparticles
Source: Nat Commun. 2025 Nov 23;16:11588. doi: 10.1038/s41467-025-66713-9 (PMC12749022; doi:10.1038/s41467-025-66713-9)
Supplement: Supplementary file 1 — Supplementary Information File [file 41467_2025_66713_MOESM1_ESM.pdf]

Supplementary Information: Wavefront Shaping of Scattering Forces Enhances Optical Trapping of Levitated Nanoparticles

Melissa Kleine[1], Michael Horodynski[2], Stefan Rotter[3], Yacine Amarouchene[1], Yann Louyer[1], Mathias Perrin[1], and Nicolas Bachelard[1]

[1]CNRS, Université de Bordeaux, LOMA, UMR 5798, Talence, France.
[2]Department of Physics, Massachusetts Institute of Technology, Cambridge, MA, 02139, USA
[3]Institute for Theoretical Physics, Vienna University of Technology (TU Wien), Vienna, Austria

November 10, 2025

Contents

1 Experimental platform **2**
1.1 Optical setup . . . 2
1.2 Wavefront shaping . . . 3
1.3 Stiffness optimization . . . 4

2 Experimental results **7**
2.1 Different cost functions . . . 7
2.2 Symmetric and asymmetric Zernike polynomials . . . 9
2.3 Trap efficiency . . . 11
2.4 Flicker noise . . . 12
2.5 Optimization performances . . . 13

3 Numerical simulations of the optimization process **14**
3.1 Forces computation and multipole expansion . . . 14
3.2 Conservative and non-conservative parts . . . 17
3.3 Optimization performances . . . 18

4 Brownian vortices **19**
4.1 Theoretical framework . . . 19
4.2 Experimental measurement of probability currents . . . 20
4.3 Experimental results . . . 20

5 Nonlinearities **22**
5.1 Duffing broadening . . . 22
5.2 Nonlinearity reduction at low pressure . . . 22
5.3 Preservation of stiffness enhancement at low pressure . . . 24

1 Experimental platform

1.1 Optical setup

Figure S1 provides a schematic description of the experimental setup. A linearly polarized 1064 nm continuous laser (AzurLight System, 10 W) delivers roughly ∼300 mW of power at the input of a vacuum chamber. Inside the chamber, the beam is focused using a high-numerical-aperture objective (Olympus LMPlan IRx100, NA = 0.8, WD = 3.4 mm) and forms a single-beam gradient optical trap, while an aspheric lens (NA = 0.55) collects the transmitted light. The particles used in the experiment consist of silica nanobeads with radii of 75, 100, 110, and 125 nm (density: $\rho = 2200$ kg.m^{-3}, refractive index: n = 1.45), sourced from *Microparticles GmbH* and *NanoCym*. Before trapping, they undergo a preheating process at 600°C for 3 hours. This treatment stabilizes the particles by eliminating Si-OH surface groups and forming durable Si-O-Si bonds[1], which ultimately densifies the nanoparticles. A suspension of these particles in isopropanol is then sprayed into the chamber at atmospheric pressure using an *Omron Micro-Air* nebulizer.

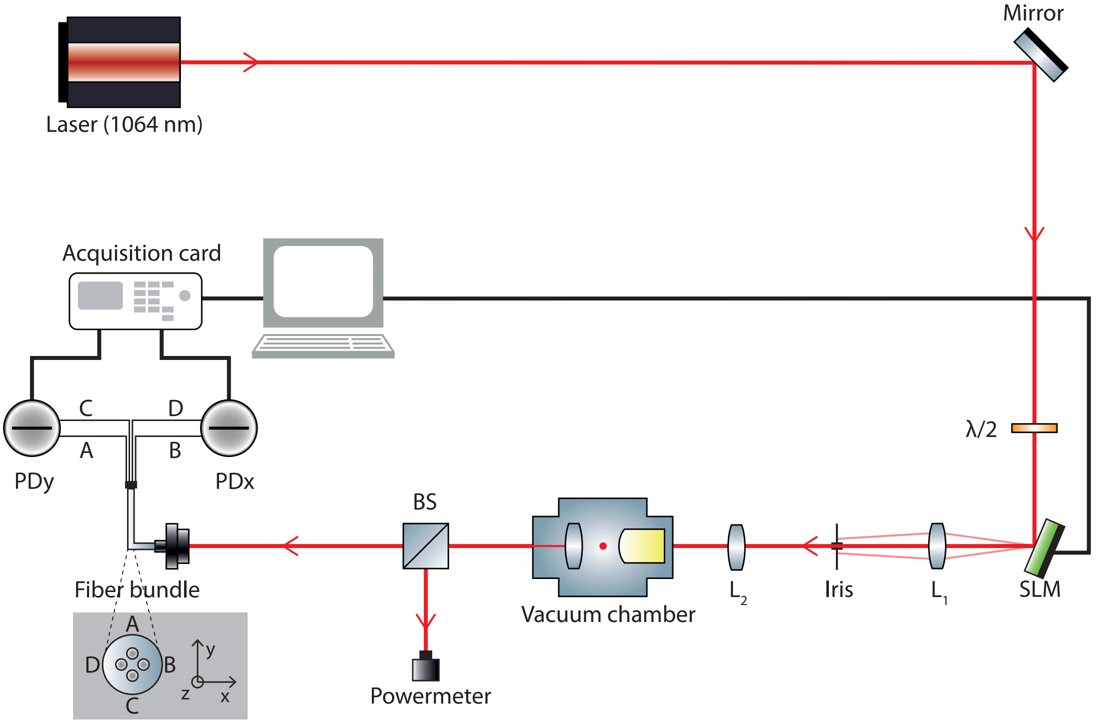

Figure S1: **Schematic representation of the experimental setup**. The optical phase of a 1064 nm continuous laser beam is spatially shaped by an Spatial Light Modulator (SLM) before being focused into a vacuum chamber using a high-numerical-aperture objective, thus forming an optical trap. The beam is then recollected and analyzed using a differential photodiode system to extract motional Power Spectral Densities (PSDs).

The center-of-mass (COM) motion of the trapped particle is detected via spatial integration of the interference pattern formed between the trapping and scattered fields. As illustrated in Fig. S1, a split detection scheme, sensitive to transverse motion, is implemented by spatially dividing the beam using a 1-to-4 multimode fiber bundle, with each fiber being coupled to a differential photodiode. Motion along the optical axis can be measured on both photodiodes. COM displacements are recorded simultaneously along all three directions using a DAQ operating at a sampling rate of 5 MS/s and

acquiring 20-second long time traces. Figure S2 provides an example of measured power spectral densities (PSDs) along all three axes under a uniform wavefront (i.e., no modulation onto the SLM).

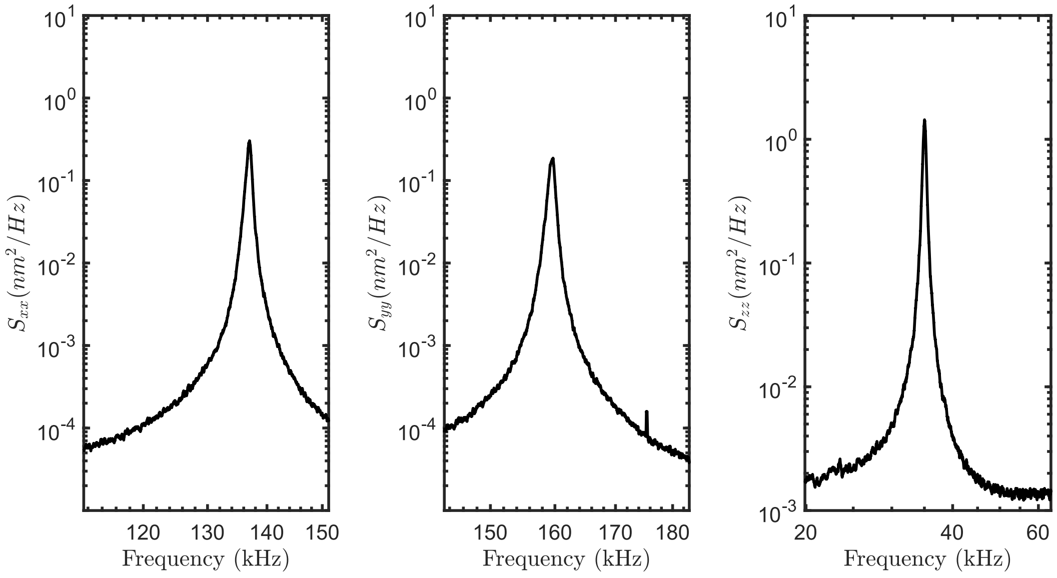

Figure S2: **Mechanical spectra measured in the absence of SLM modulation**. PSDs along all three axes for a 125 nm-radius nanoparticle, at 1 mbar, trapped using a uniform wavefront and an optical power of $\approx 300mW$ at the input of the vacuum chamber.

1.2 Wavefront shaping

The light field emitted from the laser is reflected onto a phase-only Spatial Light Modulator (SLM, Holoeye PLUTO-2.1 NIR149) with a resolution of 1920×1080 pixels and an 8 µm pixel pitch. The SLM modulates the phase of the beam before it passes through the trapping objective. Due to the Fourier transform relationship between the field at the SLM plane and the field at the focal plane of the objective, the phase-only modulation applied to the beam upstream translates (directly) into intensity modulations at the trap's location. To fully exploit the capabilities of the SLM, it is initially configured into a blazed diffraction grating. If this grating splits the beam into multiple diffraction orders, it reflects about $\approx 60\%$ of the incoming light intensity into the first order. Additional phase modulation patterns (of lower spatial frequencies) are then superimposed onto this blaze grating. According to Fourier optics, this superposition in the phase domain corresponds to a convolution of the patterns after the trapping objective, allowing the nonzero diffraction orders to be modulated. An iris is used to isolate the first diffraction order, which carries the desired phase-modulated beam, while eliminating the zero-order as well as higher-order contributions.

To estimate the position of the incident beam on the SLM, we use a masking technique combined with the diffraction grating pattern. The blaze grating pattern is applied only within a circular aperture; elsewhere, the phase modulation is set to zero (constant phase). The radius of this circular mask is chosen to be smaller than the size of the incident beam. The center of the circular mask is moved across the SLM surface in 4 pixel steps. At each position of the mask, we measure the power of the first diffraction order after the trapping objective. The recorded power indicates the overlap between the incident beam and the modulated region of the SLM. By scanning the mask position, we pinpoint the spatial location of the beam's center onto the SLM.

1.3 Stiffness optimization

The phase patterns or wavefronts used for stiffness optimization are constructed as linear combinations of Zernike polynomials. As illustrated in Fig. S3, we use a basis made of 30 polynomials, whose first 20 elements are axis-symmetric (Z_q^0), while the remaining 10 are not ($Z_q^{p \neq 0}$). Zernike polynomials are particularly well-suited for wavefront shaping due to their orthogonality and their ability to accurately represent a wide range of optical aberrations.

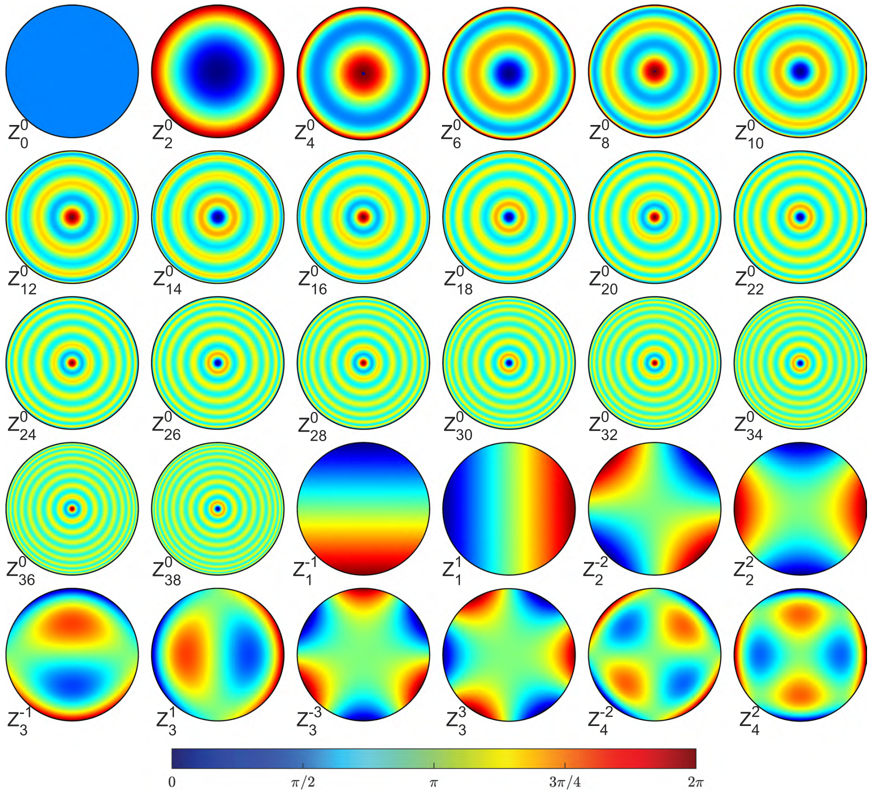

Figure S3: **Wavefront-shaping expansion basis**. Zernike polynomials used for the optimization protocol.

In order to optimize the stiffness, we employ a gradient-free simplex algorithm (*fminsearch*, Matlab). This iterative algorithm adjusts the contribution of each Zernike polynomial to maximize a given cost function without requiring gradient calculations, which are known to be extremely sensitive to experimental noise. By avoiding abrupt phase changes, this approach also ensures smooth wavefront adjustments, thus reducing the risk of particle loss throughout the process. For each iteration, an initial acquisition is performed using the uniform (i.e., unmodulated) beam to measure the baseline resonance frequencies, followed by a second acquisition with the applied phase pattern. Here, we recall that the resonance frequency $f_{i\in\{x,y,z\}}$ along each axis is related to the trap stiffness $\kappa_{i\in\{x,y,z\}}$ and the particle's mass m through the relationship

$$2\pi f_i = \sqrt{\frac{\kappa_i}{m}} \tag{1}$$

We then estimate a cost function of the form

$$f(\mathbf{X}) = \alpha(f_{opt,x}/f_{0,x})^2 + \beta(f_{opt,y}/f_{0,y})^2 + \gamma(f_{opt,z}/f_{0,z})^2 \tag{2}$$

where $f_{0,i}$ and $f_{opt,i}$ represent respectively the resonance frequencies of the uniform and optimized traps, while the terms α, β and γ stand for adjustable coefficients and $\mathbf{X}$ denotes the wavefront (i.e., linear combination of Zernike polynomials used to generate the wavefront). The coefficients

α, β and γ can be adjusted to improve the optimization of one direction over the others. This cost function is motivated by the proportionality between stiffness and the square of the resonance frequency. Measuring the uniform resonance frequency at each iteration mitigates the impact of power drifts (thus ensuring an accurate evaluation of relative stiffness improvements).

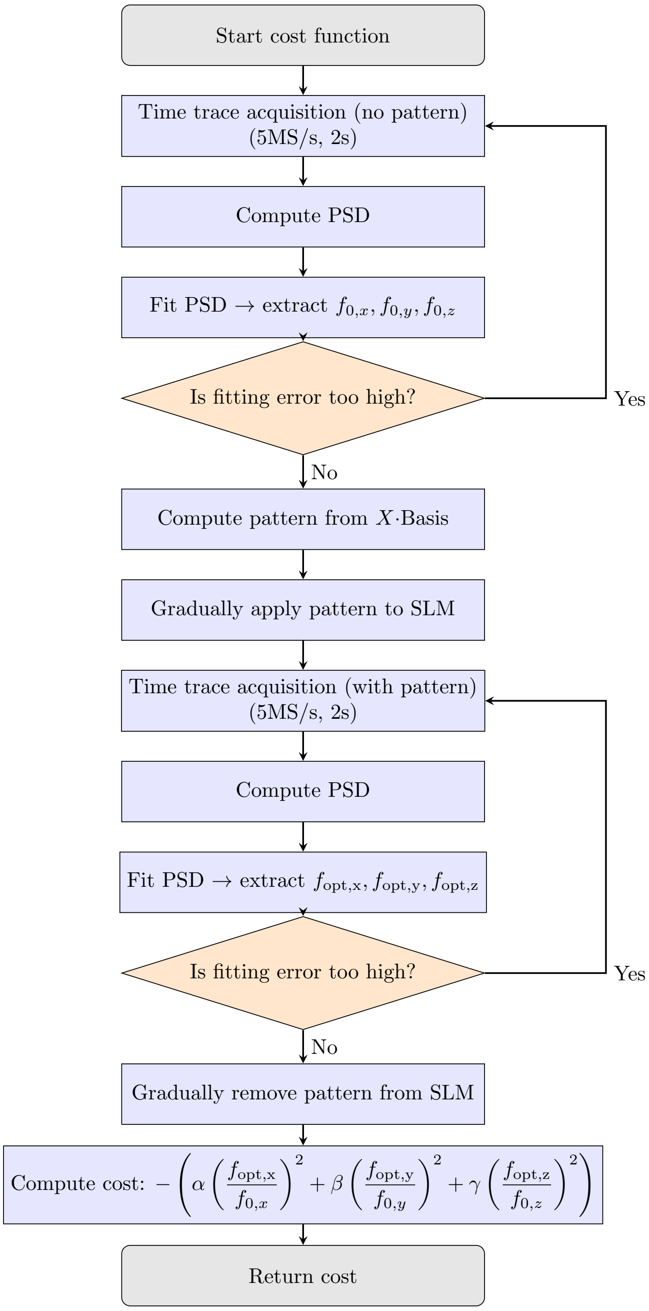

Figure S4: **Optimization block diagram**. The routine begins by acquiring a time trace without any pattern applied to the SLM (uniform profile) and computing the corresponding PSDs. The PSDs are then fitted to extract the reference trap frequencies $f_{0,x}$, $f_{0,y}$, and $f_{0,z}$. If the fitting is satisfactory, a new phase pattern, computed from the current optimization vector $\mathbf{X}$ and the Zernike basis, is gradually applied to the SLM. A second acquisition is performed and the new PSDs are fitted to extract the frequencies $f_{opt,x}$, $f_{opt,y}$, and $f_{opt,z}$. If this second fit is also acceptable, the pattern is progressively removed, and the cost function is computed using a weighted quadratic sum of frequency ratios. The value of the cost function is ultimately returned to the optimizer.

Experimentally, the cost function of equation (2) is estimated at each iteration of the optimization through the protocol described in Fig. S4. Starting from a uniform pattern displayed on the SLM, a time trace of the nanoparticle's motion is acquired for 2 seconds with a sampling rate of $5MS/s$. The corresponding PSDs along x, y and z are then computed and fitted by a lorentzian model to extract the uniform frequencies $f_{0,x}$, $f_{0,y}$ and $f_{0,z}$. If the quality of the fits does not meet the requirements, the procedure is repeated as often as needed. Then, the modulated pattern $\mathbf{X}$ provided by the algorithm is progressively applied to avoid abrupt modifications that could destabilize the trap. A time trace is collected, and the new PSDs are computed. These PSDs are fitted to extract the frequencies $f_{opt,x}$, $f_{opt,y}$ and $f_{opt,z}$. As before, this step is reproduced as long as the quality required for the fit is not reached. Ultimately, the pattern $\mathbf{X}$ is progressively removed, and the cost function is estimated using equation (2).

2 Experimental results

2.1 Different cost functions

For the experimental optimization introduced in Figure 1**b** and **c** of the main text, the black (respectively red) curves in Fig. S5 display the PSDs measured under a uniform (respectively optimized) wavefront.

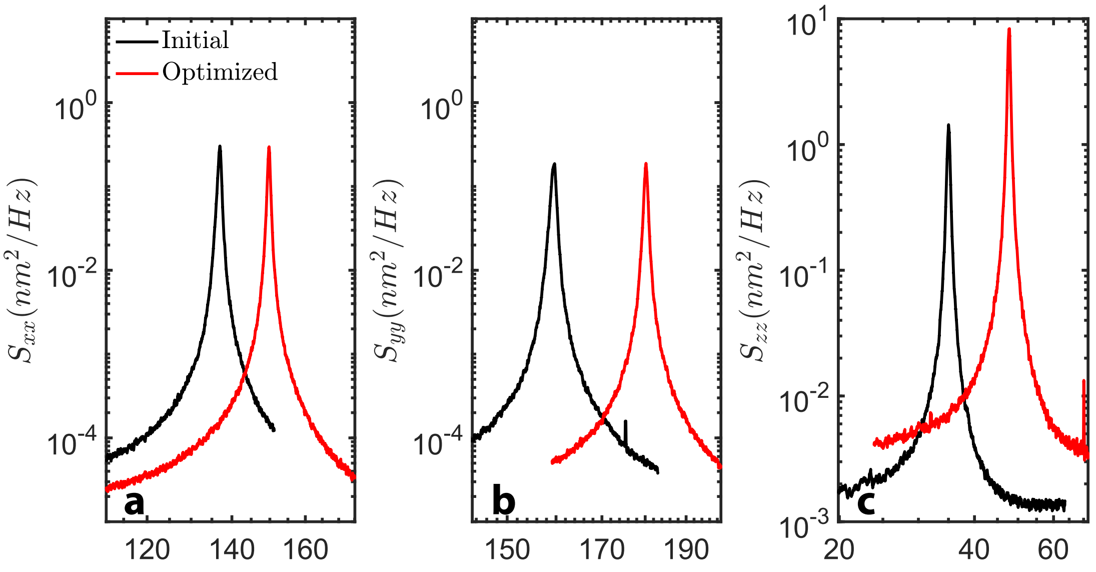

Figure S5: **Optimization of mechanical resonances**. **a**, **b** and **c**- PSDs along the x, y and z axis, respectively, which are obtained when applying the uniform (black) and optimized wavefront (red) introduced in Figure 1**b** of the main text.

The convergence of the optimization is greatly influenced by the cost function defined in equation (2). As an illustration, Fig. S6 displays the evolution of the relative stiffness throughout three optimization routines, which are performed on a particle of 110 nm in radius and using three different cost functions. The first cost function (Fig. S6**a**), optimizes the relative stiffness along all three directions and reads

$$f(\mathbf{X}) = (f_{opt,x}/f_{0,x})^2 + (f_{opt,y}/f_{0,y})^2 + \frac{1}{2}(f_{opt,z}/f_{0,z})^2$$

This results in relative stiffness ratios $\kappa_{opt,x}/\kappa_{0,x} = 1.19$, $\kappa_{opt,y}/\kappa_{0,y} = 1.26$ and $\kappa_{opt,z}/\kappa_{0,z} = 1.73$. Here, the coefficient $\gamma = 1/2$ is introduced to mitigate the influence of the enhancement along z that tends to dominate over the other directions. This cost function corresponds to the one used in Figure 1 of the main text in the case of 125 nm-radius levitated particles. The second optimization (Fig. S6**b**) focuses solely on maximizing the stiffness along the y axis, using the cost function:

$$f(\mathbf{X}) = (f_{opt,y}/f_{0,y})^2$$

Although this also improves stiffness along z, it has a negligible effect on x and the corresponding stiffness ratios are, respectively, $\kappa_{opt,x}/\kappa_{0,x} = 1.08$, $\kappa_{opt,y}/\kappa_{0,y} = 1.26$, and $\kappa_{opt,z}/\kappa_{0,z} = 1.57$. Finally, the third optimization (Fig. S6**c**) targets the x axis specifically, using the cost function:

$$f(\mathbf{X}) = (f_{opt,x}/f_{0,x})^2$$

This approach leads to the highest relative stiffness along x, making it the only case where $\kappa_x > \kappa_y$, yielding ratios $\kappa_{opt,x}/\kappa_{0,x} = 1.29$, $\kappa_{opt,y}/\kappa_{0,y} = 1.24$ and $\kappa_{opt,z}/\kappa_{0,z} = 1.79$. The three resulting

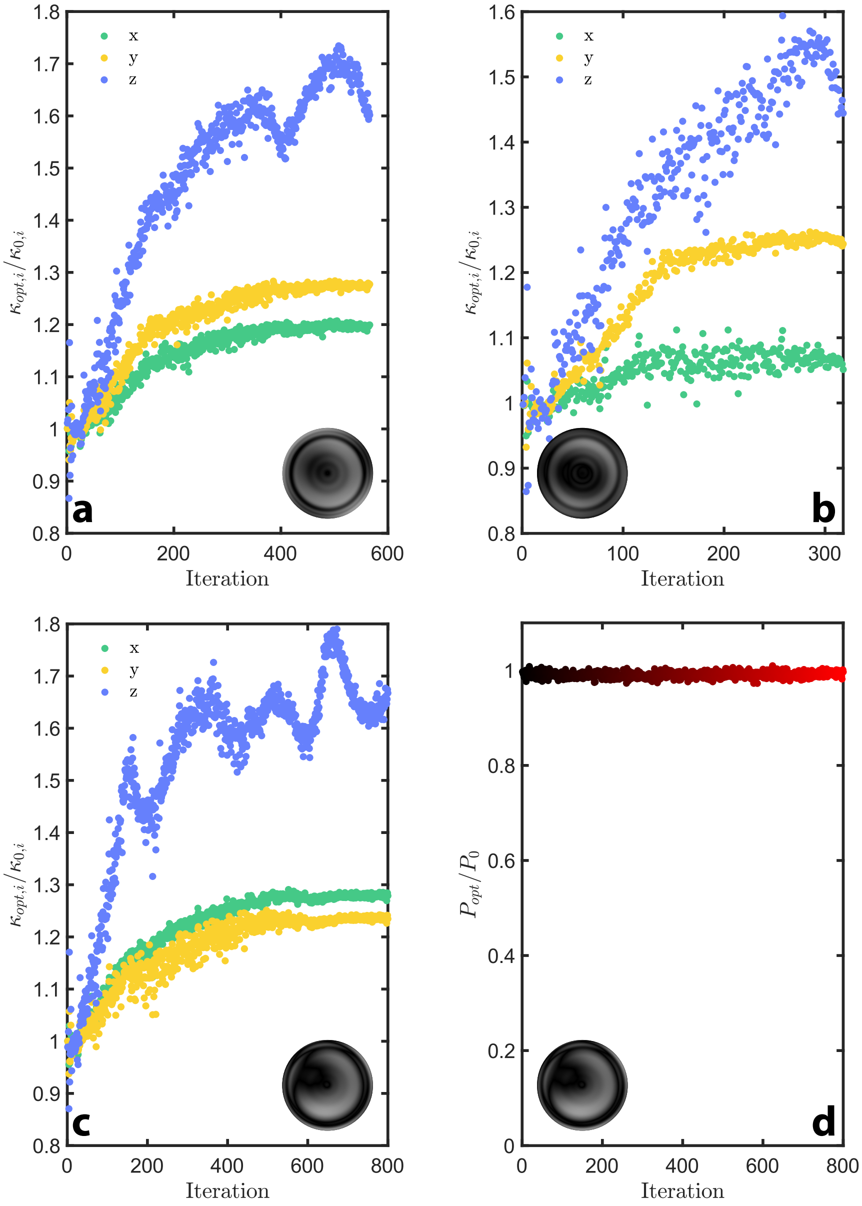

Figure S6: **Different examples of experimental optimizations**. **a, b, c**- Evolution of the relative stiffness ratios along each axis (see legend and color scheme) throughout three optimizations performed using three different cost functions. **d**- Evolution of the optical power, P_{opt}, at each iteration throughout the optimization displayed in **c**. The power is measured by a photodiode positioned after the vacuum chamber and normalized by the value collected for a uniform wavefront, P_0.

wavefronts, shown in Fig. S6, exhibit distinct differences, demonstrating the impact of the choice of the cost function on the optimized wavefront. Furthermore, Fig. S6**d** shows the evolution of the relative power measured by a photodiode located after the vacuum chamber (see Fig. S1) throughout the third optimization routine. The constant power level confirms that the optimization process neither affects the alignment of the beam nor the filling factor of the trapping objective.

2.2 Symmetric and asymmetric Zernike polynomials

Displayed in Fig. S3, the basis used to perform the wavefront expansion on the SLM is composed of axis symmetric, $Z_q^{p\neq 0}$, and asymmetric Zernike polynomials, Z_{2p}^0. The stiffness optimization being highly nonlinear and provided with multiple local minima, the stiffness improvements associated with each set of polynomials are deeply intertwined and strongly influenced by one another. Yet, experimental evidences tend to indicate that the contributions of symmetric and asymmetric polynomials are different. In Fig. S7, we levitate a nanoparticle of 75 nm in radius to which we repeatedly apply our optimization routine 8 times in a row. For each optimization, we initiate the procedure from a uniform wavefront and used the basis displayed in Fig. S3. Along the z direction, the optimization converges towards different local optima associated with stiffness enhancements $\kappa_{opt,z}/\kappa_{0,z} = [1.35, 1.37, 1.45, 1.57, 1.61, 1.40, 1.42, 1.65]$. Three of these optimizations are reported in Fig. S7**a-c**, which provide the projections of the corresponding optimized wavefronts over the Zernike basis ("Coefficients"). The vertical dashed lines set the separations between symmetric and asymmetric distributions (i.e., polynomials), while their corresponding symmetric and asymmetric contributions to the final wavefronts are provided in the insets. Despite reaching similar stiffness enhancements ($\kappa_{opt,z}/\kappa_{0,z} = 1.57$, 1.40 and 1.42), we clearly observe that the symmetric contributions differ substantially, while the asymmetric display a certain degree of similarity. To be more quantitative, in Fig. S7**d** and S7**e**, we perform the correlations between the 8 symmetric and asymmetric contributions, respectively. We report mean correlations of about 40% for symmetric patterns and around 75% for asymmetric ones. Thus, this confirm the existence of a certain "consistency" over the asymmetric patterns that are provided by the optimization, while more pronounced "variations" are reported over the symmetric ones.

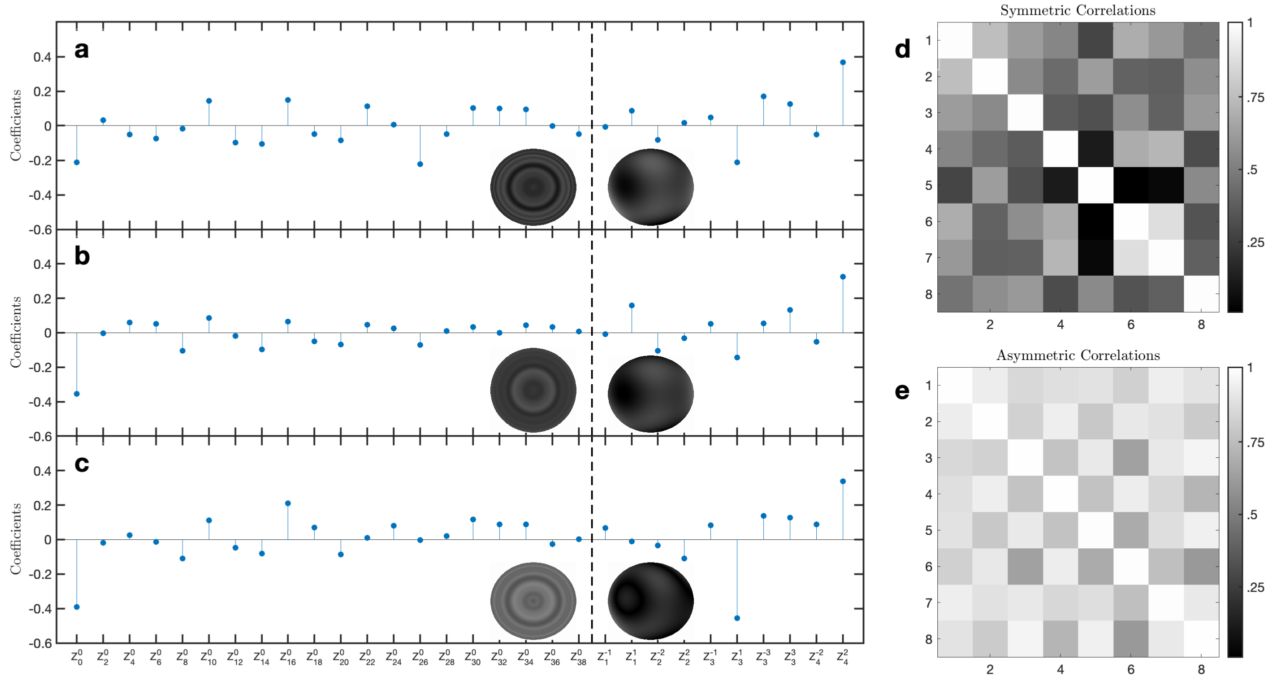

Figure S7: **Symmetric and asymmetric Zernike polynomials**. **a**, **b** and **c**- Expansion along the Zernike basis, Z_p^q, of three different wavefronts obtained by repeatedly running our optimization on the same nanoparticle (75 nm in radius). The vertical dashed line splits the axis symmetric polynomials, Z_q^0, and the asymmetric ones, $Z_q^{q\neq 0}$, while the symmetric and asymmetric components of the optimized wavefronts are provided in the insets. **d** (respectively **e**)- Correlations between the symmetric (respectively asymmetric) distributions obtained after running the optimization of **a-c** 8 times in a row.

The larger degree of correlation reported among asymmetric patterns seems to indicate that the optimization converges towards final solutions, in which certain asymmetric polynomials address systematic “errors”. In that regard, certain polynomials are probably dedicated to correct “experimental inaccuracies” (e.g., optical misalignments). Here, one can take as an example the contributions of Z_3^1, Z_3^3 or Z_4^2 in Fig. S7**a**-**c**, which seem to consistently emerge with large amplitudes to address specific optical aberrations. In contrast, the lower degree of correlation among symmetric patterns seems to indicate that these polynomials serve to converge towards local minima. Roughly speaking, one can say that these polynomials mainly focus on providing a ”physical” improvement of the stiffness as opposed to improvements achieved through corrections of “experimental inaccuracies”. Nonetheless, it is important to notice that this behavior is is not unique to symmetric polynomials. Indeed, some asymmetric polynomials can display strong variations that could be indicative (like in the symmetric case) of a ”physical” stiffness improvement. Here, one can take as an example the contributions of Z_1^1 or Z_4^{-2} in Fig. S7**a**-**c**, whose coefficients can flip sign to provide opposed contributions to the final wavefront.

2.3 Trap efficiency

To demonstrate that the optimization achieves more photon-efficient optical trapping (i.e., same resonance frequency for less power), we provide in Fig. S8 the experimental results of an optimization performed on a nanoparticle of 125 nm in radius. This optimization is performed initially for a power emitted by the laser of 590 mW. Afterwards, the power is varied to confirm that the stiffness enhancement is indeed independent of laser power. Owing to losses along the optical setup, the laser power does not correspond to the actual power focused onto the particle (that sets closer to 100 mW). When the lasing power is varied, Fig. S8**a** displays in green, yellow and blue the enhancements that are measured along x, y and z, respectively. The vertical red dashed line marks the power P_{opt} at which the optimization is performed. Here, constant stiffness enhancements of $\kappa_{opt,x}/\kappa_{0,x} \approx 1.35$, $\kappa_{opt,y}/\kappa_{0,y} \approx 1.4$ and $\kappa_{opt,z}/\kappa_{0,z} \approx 2$ are reported regardless of optical power. The larger imprecision along z originates from the fact that the mechanical resonance along that direction is broad spectrally (lower optical confinement and quality factor). This induces a certain imprecision in the tracking of the resonance frequency, f_z, which translates into larger fluctuations on the estimation of the stiffness enhancement $\kappa_{opt,z}/\kappa_{0,z}$. This panel clearly emphasizes that the optimization is perfectly independent of optical power.

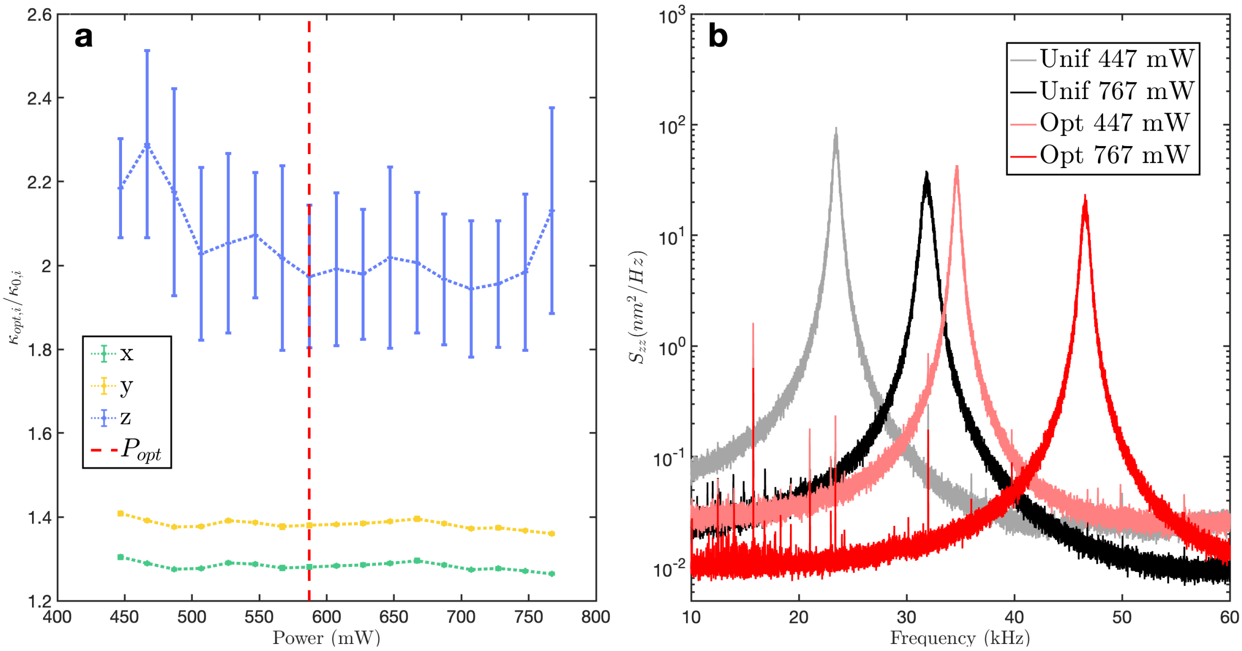

Figure S8: **Variation of optical power**. **a-** A 125 nm-radius nanoparticle is optimized using a power emitted by the laser of 590 mW (vertical red dashed line, P_{opt}). Afterwards the laser power is varied from 447 mW to 767 mW. The enhancements $\kappa_{opt,x}/\kappa_{0,x}$,$\kappa_{opt,y}/\kappa_{0,y}$ and $\kappa_{opt,z}/\kappa_{0,z}$ are displayed in green, yellow and blue, respectively, and appear independent of optical power. **b-** For the optimization performed in **a**, the thick and light black (respectively red) curves display the mechanical spectra, S_{zz}, obtained for a uniform (respectively optimized) wavefront and for laser powers of 447 mW and 767 mW, respectively.

Then, Fig. S8**b** focuses on the stiffness enhancement of ≈ 2 that is achieved along z. We expect that the optimized profile will provide the same frequency as in the uniform case but with twice less power. We plot in thick black and thick red, the PSDs that are measured, respectively, under a uniform and an optimized wavefront when the optical power is set to 767 mW. We plot in light dark and light red, the PSDs that are measured, respectively, under a uniform and an optimized wavefront when the optical power is set to 447 mW. For experimental reasons, we only achieved here a reduction of lasing intensity by a factor 1.7, which corresponds to the optimized resonance moving from 47 kHz down to

34.5 kHz (i.e., a reduction by a factor of $\approx \sqrt{1.8}$). Yet, we see that the optimized resonance using a power of 447 mW (34.5 kHz) almost matches the uniform one obtained for 767 mW (32 kHz). Simple calculations confirm that both curves would align perfectly when using 767/2=383 mW. Therefore, we experimentally confirm that power can be substantially reduced with our method.

2.4 Flicker noise

Flickering is known to be inherent to different technologies of modulators such as digital liquid-crystal SLMs (like the device used in this work) or Digital Micromirror Devices (DMDs). There, flicker noise originates from the fact that each pixel is addressed using a binarized voltage and materializes, in the spectral domain, by the presence of multiple harmonics of the refreshing rate. As shown in [2], the device used throughout this work (Holoeye, Pluto-2.1 NIR 149), has been adapted to display reduced flicker noise. It is characterized by a maximum peak-to-peak phase fluctuation of about 1% and harmonics constrained below a few kHz. We reproduce in Fig. S9 the PSDs provided in Figure 1**b** of the main text. Here, both the uniform (black) and optimized PSD (red) are plotted over a broad spectral range and display 60 Hz-harmonics up to 2 kHz. Even though many harmonics are observed, they are ultimately spectrally narrow, such that their cumulative contribution remains negligible compared to the informative signal (i.e., mechanical resonances). More importantly,the flicker harmonics (below 2 kHz) do not overlap with the mechanical resonances (above 20 kHz) and thus have no influence onto them. At last, flicker noise can be totally removed by using flicker-free SLM technologies. For instance, analog liquid-crystal SLMs, which address individual pixels with analog voltage, are commercialized by mainstream companies and provide only residual flicker noise[3].

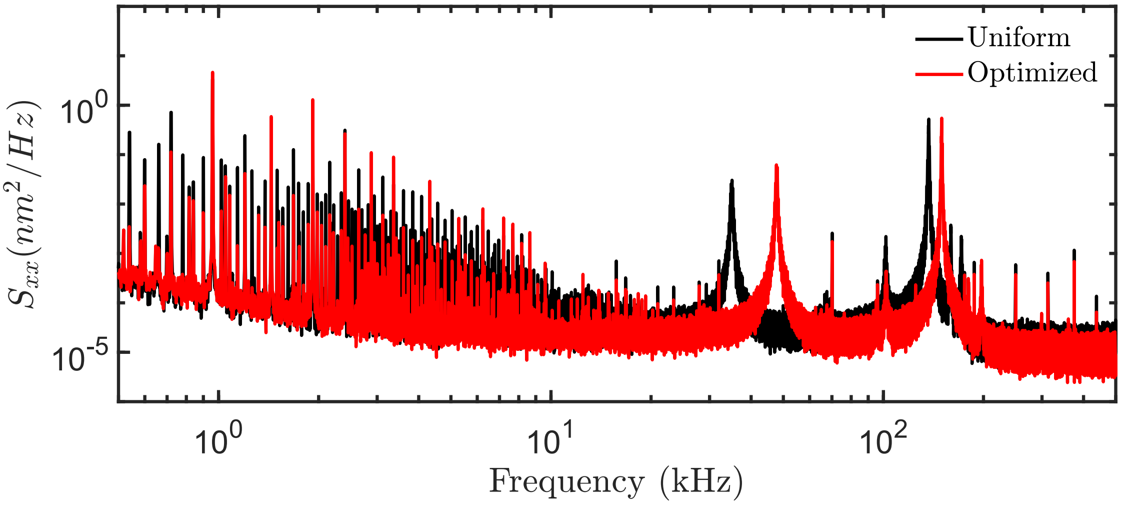

Figure S9: **Flicker noise**. Unsmoothed PSDs recorded after the optimization introduced in Figure 1**b** and **c** of the main text. The black and red curve are recorded for a uniform and optimized pattern, respectively. Below $\approx$ 2 kHz, both spectra display 60 Hz harmonics characteristic of flicker noise.

2.5 Optimization performances

The overall optimization process takes between 200 to 500 iterations, which translates into roughly 2 to 5 hours. Yet, we would like to stress here that no effort was provided to speed up the routine. We are convinced that the procedure could probably be reduced to a few minutes or below. For instance, the acquisition time and the data transfer can be strongly shorten, while switching from one wavefront to the next can also be substantially speed up.

The optimization possesses multiple local minima, which lead to different final wavefronts as well as a dispersion in the final enhancement ratios that one can reach. This point is highlighted in Fig. S7, in which we run the protocol 8 times consecutively on a 75 nm-radius particle. There, we measure variations of $\kappa_{opt,x}/\kappa_{0,x}$ from 1.13 to 1.31, $\kappa_{opt,y}/\kappa_{0,y}$ from 1.17 to 1.32, and finally $\kappa_{opt,z}/\kappa_{0,z}$ from 1.35 to 1.65. Thus, final enhancement ratios can substantially fluctuate depending on the local minimum that is reached.

When the optimization has converged, the corresponding optimized wavefront reveals stable over weeks. For instance, for the experiment displayed in Fig. S10**b**, we run the optimization on a nanoparticle of radius 125 nm. Over the course of two weeks, this pattern was successively applied to particles of different radii. This figure shows the relative stiffness enhancements for particles of different radii using the optimized pattern obtained in Figure 1 of the main article. These results indicate that a pattern optimized for a given size remains effective for particles of different radii. Nonetheless, the optimization is less efficient than when performed directly on the particle of the proper radius (see Fig. S10**a** which reproduces Figure 1d of the main text for comparison).

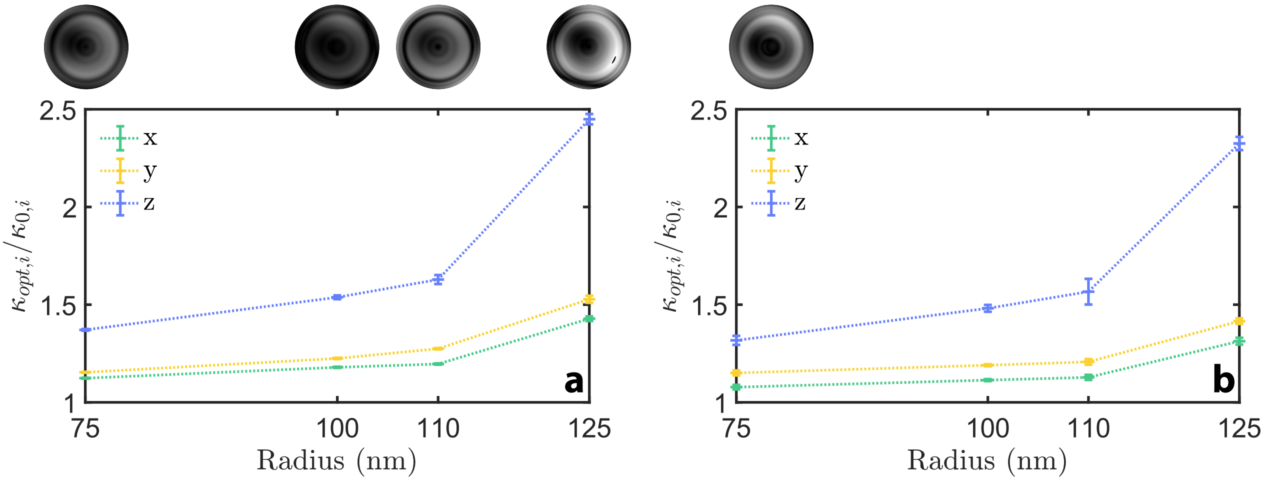

Figure S10: **Stiffness ratios obtained for different radii**. **a-** (Figure 1**d** of the main text). The green, yellow and blue curve display respectively the enhancements $\kappa_{opt,x}/\kappa_{0,x}$, $\kappa_{opt,y}/\kappa_{0,y}$ and $\kappa_{opt,z}/\kappa_{0,z}$ obtained when running the optimization onto particles of different radii. The corresponding wavefronts obtained for each optimization are reported in the upper part of the panel. **b-** The optimization is performed on a 125 nm-radius particle. The obtained wavefront is displayed in the upper part of the panel and applied to particles of radius 75, 100 and 110 nm in radius. Same color code as in **a**. In panels **a** and **b**, for each radius, the error bars characterize the fluctuations of the final ratios under their respective optimized wavefront.

3 Numerical simulations of the optimization process

3.1 Forces computation and multipole expansion

To model the trapping field for various numerical apertures, NA, filling factors, f_0, and wavefronts, $\mathbf{X}$, we use a modified Debye integral. Specifically, we include a thin-lens apodization function (see equation (3.56) of Ref [4]), which accounts for the SLM-modulated wavefront. The field scattered by the particle is expressed using the Generalized Lorentz Mie Theory (GLMT), which ultimately enables to compute 'exactly' the optical forces using the Maxwell Stress Tensor (MST) [5]. This method is used to benchmark a much faster semi-analytical method, which provides the total force as a sum of different multipole contributions [6, 7]. Even for dielectric objects, these contributions decompose into electric multipoles (e.g., dipole, quadrupole) produced by field-induced charge displacements, and magnetic multipoles originating from corresponding electric-current loops.

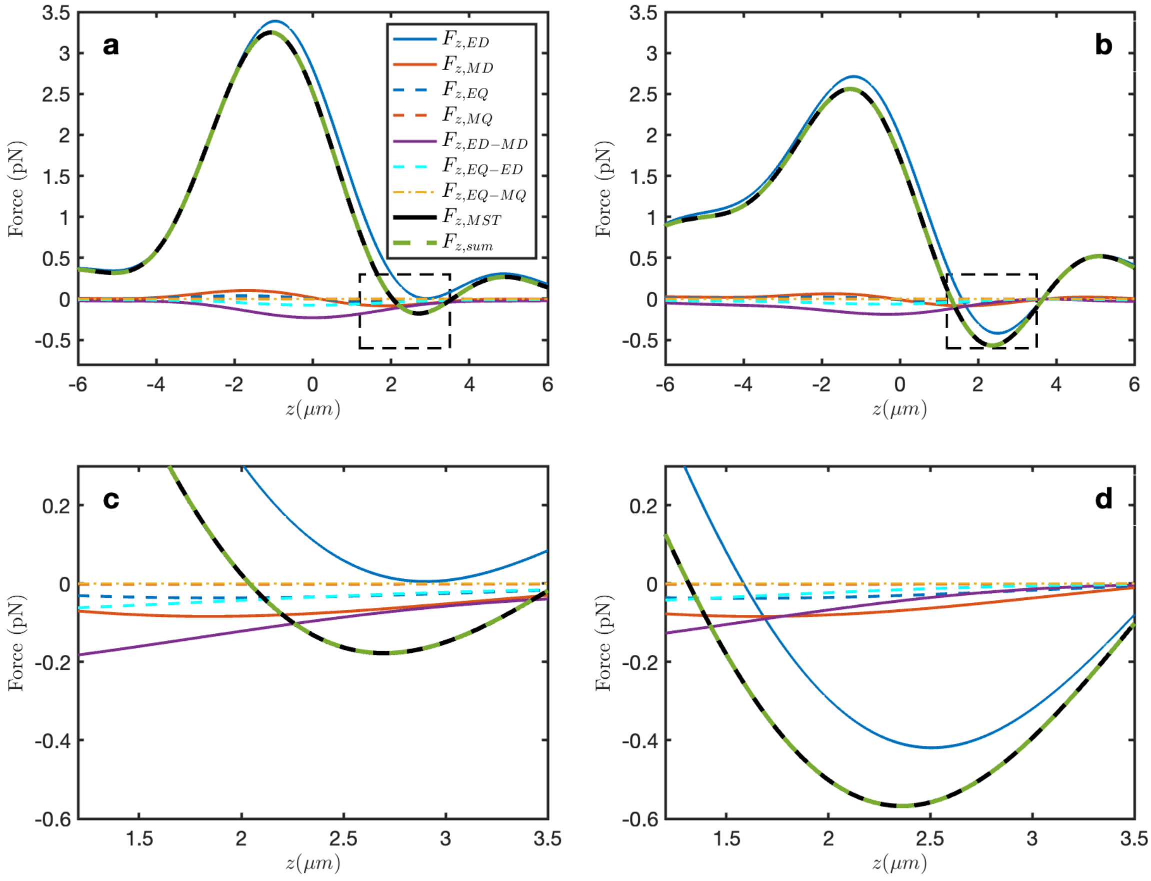

Figure S11: **Simulations of axial-force landscapes**. **a**- Landscape of the axial force, F_z, for a uniform wavefront. The black curve represents the 'exact' MST computation, $F_{z,MST}$. The other curves (see legend and text) describe the different multipole contributions. When adding up all these contributions, we obtain a total force (dashed green, $F_{z,sum}$) identical to the one computed using the MST. **b**- Similar force landscape to that in **a** but here for an optimized wavefront (same legend and color code). **c** and **d** show respectively magnified views of **a** and **b** around their corresponding trapping points (dashed black frames).

The optimization routine is performed by computing, at each iteration, the force landscapes along x, y and z, labeled $F_x(x)$, $F_y(y)$ and $F_z(z)$, respectively. Such computations are achieved using the multipole method (introduced above) up to the quadrupole order. The modulated wavefront is reproduced by decomposing the apodization function into 32 to 128 concentric rings, in which the optical phase is uniform. The distribution of the phase on each ring emulates a wavefront $\mathbf{X}$, which is

iteratively optimized to maximize a cost function similar to the one provided in section 1.3. Varying the number of rings, as well as the starting guess, we observe a convergence towards wavefronts similar to the ones obtained experimentally. Figure S11 shows in black the axial-force landscape, $F_{z,MST}(z)$, computed for a uniform (panel **a**) and an optimized wavefront (panel **b**), which display a magnification in axial stiffness κ_z close to a factor of 2.2 (see Figure 2 of the main text). Zoomed-in views of both landscapes are provided in Figs. S11**c** and **d**. Here, the simulation parameters are set to an NA=0.68 and a filling factor $f_0 = 0.7$, for a particle of radius 125 nm trapped using a beam power of 350 mW. We underline, however, that a stiffness magnification higher than 2 (as discussed in this work) has been observed numerically for a wide range of particle radii, numerical apertures and filling factors (see section 3.3). In the optimized case, the focal point is shifted along the z direction. To take that modification into account and locate the new focal point, the z coordinate has been shifted such that the conservative part of the force (i.e., gradient force $F_{g,opt}$, see section 3.2) is zero when the new coordinate reaches 0.

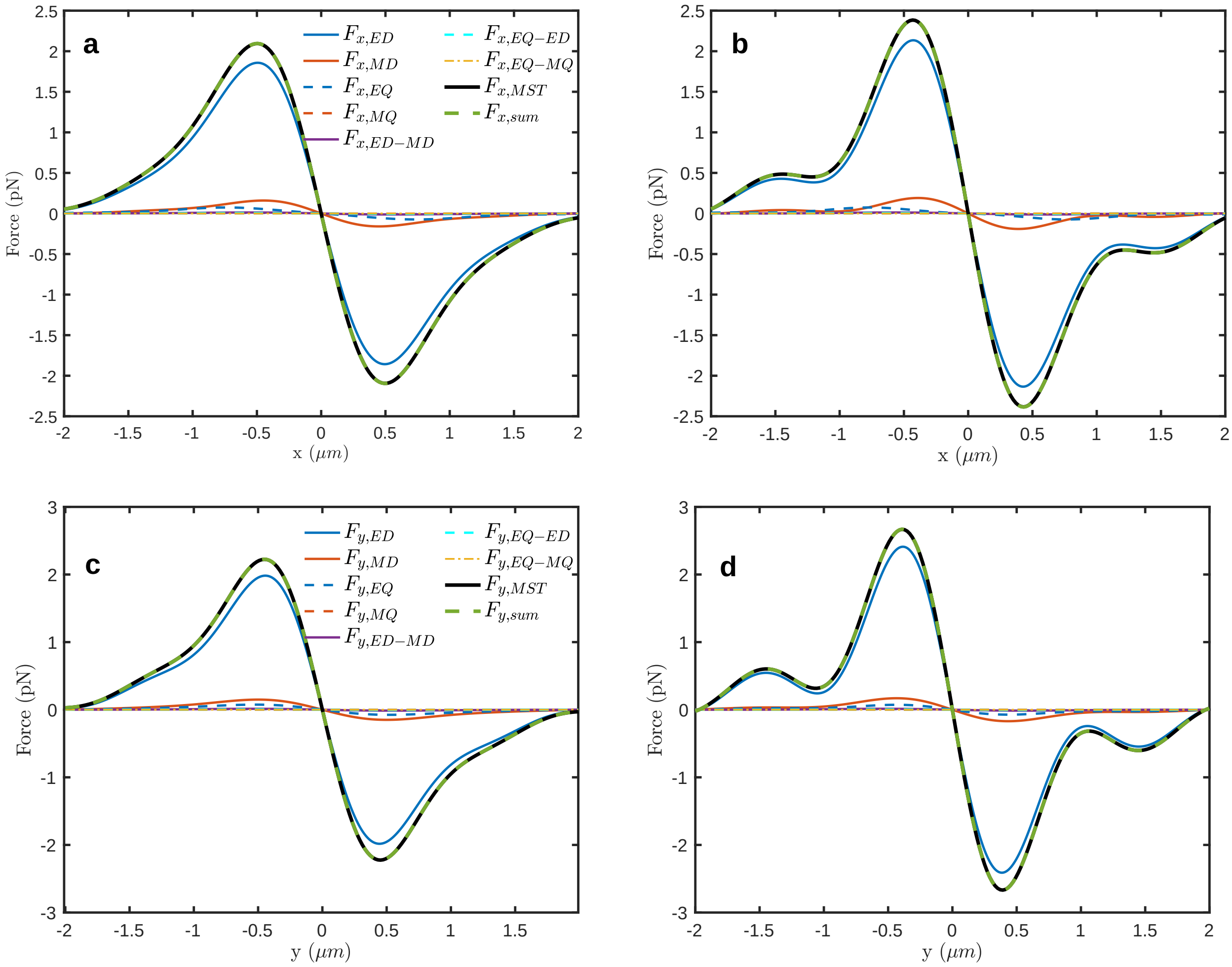

Figure S12: **Simulations of transverse-force landscapes**. **a**, **b**- Force landscapes F_x computed using the two approaches introduced in Fig. S11 and for the same wavefronts (respectively uniform in **a** and optimized in **b**). **c**, **d**- Force landscapes F_y computed using the two approaches introduced in Fig. S11 and for the same wavefronts (respectively uniform in **c** and optimized in **d**).

In Fig. S11**a** and **b**, the different multipole contributions provided by our numerical method are displayed. In particular, we plot the force originating from the electric dipole (ED, blue), the magnetic dipole (MD, red), the electric quadrupole (EQ, dashed blue) and the magnetic quadrupole (MQ, dashed red). There, we also report the force produced by the interferences between the electric and magnetic

dipoles (ED-MD, purple), between the electric dipole and quadrupole (ED-EQ, dashed light blue) as well as between the electric and magnetic quadrupoles (EQ-MQ, dot-dashed yellow) [6]. The curve in dashed green shows the sum of these different multipole contributions, which matches the 'exact' computation performed using the Maxwell Stress Tensor (black). In Figure 2**a** and **b** of the main text, the interference terms have been added to the magnetic dipole and electric quadrupole contributions to make these figures easier to understand. As expected for such a small nanoparticle, these simulations clearly emphasize that the electric dipole is the dominant contribution to the total force along the z-axis. They also show that the optimization mainly acts on the electric-dipole term, while other contributions remain largely unaffected. In other words, the optimization primarily reshapes the electric-dipole contribution in order to increase the stiffness.

For the optimization displayed in Figure 2 of the main text and reproduced in Fig. S11, Fig. S12 shows in black the exact calculations of the forces along the two transverse directions, respectively $F_{x,MST}$ and $F_{y,MST}$. We also provide the multipole expansion of the forces using the same color code and naming scheme as in Fig. S11. Similarly to the axial direction, we observe that the optimization mainly acts on the dominant electric dipole to improve the stiffness along the transverse directions. As sketched in Figure 2c of the main text, the optimization brings the particle closer to the focus, where the intensity gradient is stiffer in the transverse plane, which readily improves the optical confinement.

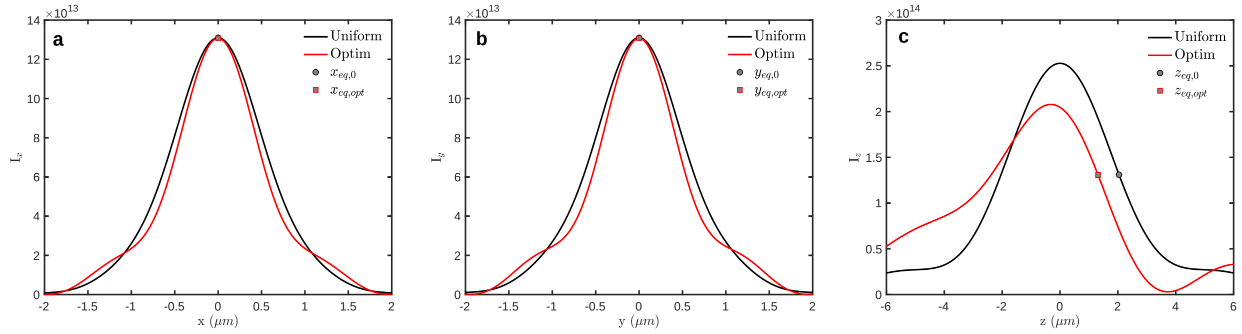

Figure S13: **Uniform and optimized intensity distributions close to equilibrium**. **a**, **b** and **c**- Intensity profiles I_x, I_y and I_z (in arbitrary units), which are computed respectively along x, y and z for the uniform (red) and optimized (black) wavefront of Fig. S11, respectively. The black squares and red dots pinpoint the equilibrium location along each axis.

We show experimentally in Fig. S8 that the optimized trap becomes more photon-efficient, in that it can produce the same stiffness as in the uniform case but with significantly less incoming laser intensity. This point is numerically confirmed in Fig. S13, which plots along the three directions x, y and z the field intensities in the vicinity of the focal spot for the uniform (black) and optimized (red) wavefronts. We readily observe that the intensity at the equilibrium position remains similar in both cases (black squares and red dots for uniform and optimized z_{eq}, respectively). Thus, as the stiffness is more than doubled by the optimized wavefront (multiplied by ≈ 2.2), one can achieve the same stiffness as in the uniform case with an incoming laser power reduced by more than 50% (i.e., divided by a factor of 2.2).

3.2 Conservative and non-conservative parts

The multipole expansion performed in Fig. S11 and S12, can be harnessed to decompose analytically the different terms into their non-conservative (i.e., scattering) and conservative (i.e., gradient) parts [8]. Since this decomposition is only provided along z in Figure 2**d** of the main text, we only consider the axial forces below. In particular, the conservative and non-conservative parts are labeled respectively $F_{g,j}$ and $F_{s,j}$, with j indicating the multipole considered. For the conservative parts:

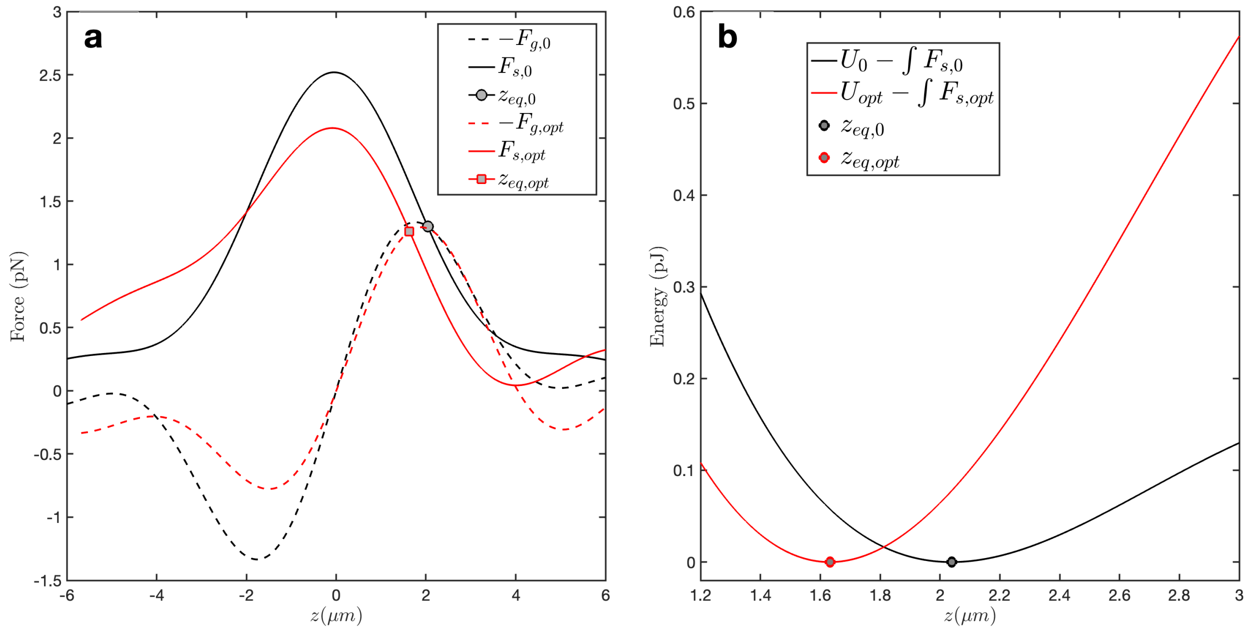

Figure S14: **Conservative and non-conservative contributions to the axial force**. **a-** The total axial force, F_z, is decomposed into its conservative and non-conservative parts, see equations (3, 4). For the uniform wavefront, we plot in black $-F_{g,0}$ and $F_{s,0}$, which intersect at the equilibrium position $z_{eq,0}$. For the optimized wavefront, we plot in red $-F_{g,opt}$ and $F_{s,opt}$, which intersect at the equilibrium position $z_{eq,opt}$. **b-** For the uniform (respectively optimized) wavefront used in Figure 2d of the main text, the black (respectively red) curve displays the optical potential $U_0(z)$ (respectively $U_{opt}(z)$) from which the work of the scattering force $F_{s,0}$ (respectively $F_{s,opt}$) is subtracted. The minima $z_{eq,0}$ and $z_{eq,opt}$ mark the equilibrium positions when the uniform and the optimized wavefront are applied, respectively.

$$\begin{aligned} F_{g,ED} &= \frac{\varepsilon_0}{2}\mathrm{Re}\left(\alpha_{\mathrm{ED}}\right).\mathrm{Re}\left(\frac{\partial \mathrm{E_j}}{\partial \mathrm{z}}\mathrm{E_j^\star}\right) \\ F_{g,MD} &= \eta_0^2\frac{\varepsilon_0}{2}\mathrm{Re}\left(\alpha_{\mathrm{MD}}\right).\mathrm{Re}\left(\frac{\partial \mathrm{H_j}}{\partial \mathrm{z}}\mathrm{H_j^\star}\right) \end{aligned} \quad (3)$$

while, for the non-conservative parts:

$$\begin{aligned} F_{s,ED} &= \frac{\varepsilon_0}{2}\mathrm{Im}\left(\alpha_{\mathrm{ED}}\right).\mathrm{Im}\left(\frac{\partial \mathrm{E_j}}{\partial \mathrm{z}}\mathrm{E_j^\star}\right) \\ F_{s,MD} &= \eta_0^2\frac{\varepsilon_0}{2}\mathrm{Im}\left(\alpha_{\mathrm{MD}}\right).\mathrm{Im}\left(\frac{\partial \mathrm{H_j}}{\partial \mathrm{z}}\mathrm{H_j^\star}\right) \\ F_{s,ED-MD} &= -\frac{k^4}{12\pi\varepsilon_0 c}\mathrm{Re}\left(\alpha_{\mathrm{ED}}\ \alpha_{\mathrm{MD}}^\star.\left[\mathbf{E}\wedge\mathbf{H}^\star\right]\cdot\mathbf{u_z}\right), \end{aligned} \quad (4)$$

where $j \in [x, y, z]$ and summation over repeated indices is implied. Here, η_0 stands for the vacuum impedance, k for the wavenumber, and $(\mathbf{E}, \mathbf{H})$ refer to the trapping field (uniform or optimized). At

last, $\alpha_{ED,MD}$ stand respectively for the complex polarizabilities of the electric and magnetic dipoles, while $\mathbf{u_z}$ corresponds to the unitary vector along z.

The conservative, F_g, and non-conservative part, F_s, of the total force F_z are obtained by summing the different expressions provided respectively on equation (4) and (5). Note that, as the quadrupole terms make only small contributions to the total force in the present case (see Fig. S11), we can safely assume that they do not affect the mechanism at play. As a result, we can indifferently incorporate their contributions onto either F_s or F_g (here, we chose the former). Figure S14**a** displays $-F_g$ (dashed) and F_s (solid) for a uniform (black, subscript 0) and an optimized wavefront (red, subscript *opt*). The intersection of both curves defines the equilibrium position, z_{eq}. We clearly observe that, after the optimization, the conservative force F_g remains almost identical in the vicinity of the equilibrium position. In sharp contrast, we observe that the non-conservative force F_s is largely shifted towards the focus. From this observation, we deduce that the optimization is more efficient when there is a substantial scattering force to reduce. This emphasizes that our approach should reveal particularly efficient in the context of atoms trapped in optical tweezers, which can display large scattering cross sections near resonance[4].

Alternatively, one can understand the improvement of the stiffness by considering the deformation of the "effective" potential associated with the optical forces. This potential can be defined by subtracting the work of scattering force, $\int^z F_s(u)du$, from the potential associated to the gradient force, $U(z)$. Figure S14**b** displays in black (respectively red) the effective potential computed under a uniform (respectively optimized) wavefront. We clearly observe that this "effective" potential is shifted towards focus by the optimization, while displaying a stronger confinement. Moreover, it appears more quadratic and therefore less prone to Duffing nonlinearities (see section 5.1).

3.3 Optimization performances

We used our numerical model to estimate the highest order of Zernike polynomials (see Fig. S3) that should effectively have a substantial influence on the optimization. In Fig. S15**a**, we run the optimization while progressively increasing the number of rings that are used to discretize the optical phase. For a 5-ring discretization, the optimization is only partial and the optimal solution is reached above 15 rings. As a result, Zernike polynomials exhibiting a faster modulation (more rings) are likely to be irrelevant to the optimization. From Fig. S3, one can conclude that the symmetric polynomials Z_{32}^0 to Z_{38}^0 are likely to have almost no contribution to the optimization.

Next, we harness our model to estimate the largest stiffness enhancement achievable. We explained in section 3.2 that our mechanism relies on the fact that we are able to reduce the contribution of the scattering force, while keeping the gradient force almost unchanged. In short, the optimization relies on the ability to reduce the role of the scattering force that tends to move the particle away from focus. In that sense, the stronger the scattering force, the better our approach operates. Thus, since larger particles display larger scattering cross sections, they provide stronger scattering forces and the optimization should work even better. At this point, we need to stress that the size of the particle one can levitate with a given optical setup is limited by the numerical aperture of the objective (NA). Typically, one can levitate nanoparticles up to a few hundreds of nm using NA from .7 to .8. To levitate larger particles (e.g., micronsize particles [9]) one must rely on smaller NA. We provide in Fig. S15**b** numerical simulations mimicking our experimental setup. Specifically, we run the optimization on a silica nanoparticle while varying its radius. As stated above, the optimization clearly improves with the size (and thus the scattering cross section). In our current optical setup (i.e., when considering a NA of 0.8), these simulations show that trapping becomes instable for radii exceeding 140 nm, while an enhancement of almost 6 is predicted for particles of radius 135 nm. We are also convinced that this technique can be straightforwardly transferred to optimize micronsize particles levitated using lower NAs. For larger particles, the presence of multiple electric and magnetic dipoles provides a deeper controllability over the light field[10], which can be harnessed to design strong optical confinements. As such, we anticipate that our approach should reveal even more efficient in the context of larger levitated objects.

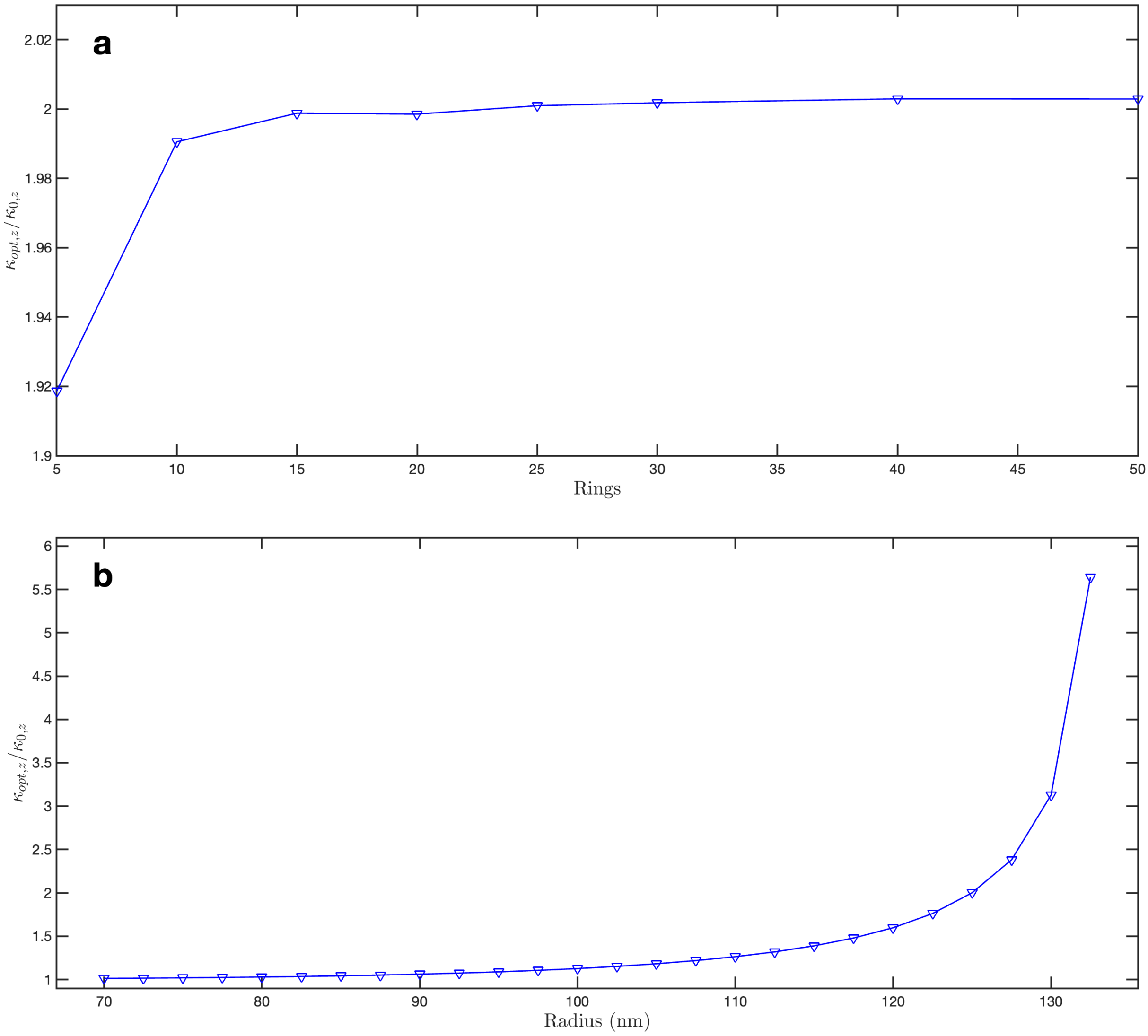

Figure S15: **Optimization performances**. **a-** Numerical optimization performed for different spatial resolutions. Evolution of the stiffness enhancement, $\kappa_{opt,z}/\kappa_{0,z}$, of a nanoparticle (125 nm in radius) when varying the spatial resolution of the wavefront (i.e., number of sampling rings). **b-** Stiffness enhancement for various nanospheres' radii. The numerical model used in Figure 2 of the main text is adapted to optimize the stiffness ratio $\kappa_{opt,z}/\kappa_{0,z}$ while varying the radius of the silica nanoparticle. The enhancement improves as the radius of the particle—and therefore its scattering cross section—increases. For radii larger than 140 nm, the trap becomes unstable.

4 Brownian vortices

4.1 Theoretical framework

In liquids, non-conservative scattering forces in optical traps are known to give rise to non-equilibrium probability currents, commonly referred to as Brownian vortices[11]. These currents have also been reported for trapped particles governed by underdamped motions, as demonstrated in both theoretical[12] and experimental studies[13].

We denote $P(\mathbf{x}, \mathbf{v}, t)$ the probability distribution in position and velocity space ($\mathbf{x}$ and $\mathbf{v}$, respectively) of a nanoparticle over time, t. The evolution of this probability distribution is governed by a

Fokker-Planck equation

$$\frac{\partial P(\mathbf{x}, \mathbf{v}, t)}{\partial t} = -\nabla_{\mathbf{x}} \mathbf{J}_{\mathbf{x}}(\mathbf{x}, \mathbf{v}, t) - \nabla_{\mathbf{v}} \mathbf{J}_{\mathbf{v}}(\mathbf{x}, \mathbf{v}, t) \tag{5}$$

in which $\mathbf{J}_{\mathbf{x}}$ and $\mathbf{J}_{\mathbf{v}}$ stand for the space and velocity probability currents, respectively. These currents fulfill

$$\begin{aligned} \mathbf{J}_{\mathbf{x}} &= \mathbf{v}P(\mathbf{x}, \mathbf{v}, t) \\ \mathbf{J}_{\mathbf{v}} &= -\frac{k_B T \gamma}{m^2} \nabla_{\mathbf{v}} P(\mathbf{x}, \mathbf{v}, t) - \frac{\gamma}{m} P(\mathbf{x}, \mathbf{v}, t) + \frac{1}{m} \mathbf{F}_t(\mathbf{x}) P(\mathbf{x}, \mathbf{v}, t) \end{aligned}$$

where γ stands for the friction coefficient, m the particle's mass, $\mathbf{F}_t$ the total force acting on the particle, k_B the Boltzmann constant, and T the temperature. Derivations based on a minimal scattering model (MSM) with a Gaussian field distribution indicate that the amplitude of Brownian vortices is directly affected by the distribution of the scattering force[12]. Thus, a change in the probability currents is indicative of a change in the scattering-force landscape.

4.2 Experimental measurement of probability currents

Experimentally, the probability $P(\mathbf{x}, \mathbf{v}, t)$ is estimated using the following expression

$$P(\mathbf{x}, \mathbf{v}, t) = \langle \delta(\mathbf{x} - \mathbf{X}_t) \delta(\mathbf{v} - \mathbf{V}_t) \rangle \tag{6}$$

where $\langle . \rangle$ denotes the statistical average, while $\mathbf{X}_t$ and $\mathbf{V}_t$ represent the instantaneous position and velocity of the particle at time t, respectively. The effective probability currents are then given by

$$\begin{aligned} \overline{\mathbf{J}_{\mathbf{x}}} &= \langle \mathbf{V}_t \delta(\mathbf{x} - \mathbf{X}_t) \rangle \\ \overline{\mathbf{J}_{\mathbf{v}}} &= \langle \dot{\mathbf{V}}_t \delta(\mathbf{v} - \mathbf{V}_t) \rangle \end{aligned} \tag{7}$$

These effective currents can be accurately determined in the underdamped regime from temporal traces of the nanoparticle's position using standard conditional binning histograms. The photodiodes produce electric signals, $V(t)$, which relate to the nanoparticle's motion, $x(t)$, through a calibration factor C_{calib} (in V/m) fulfilling in the spectral domain $S_{VV}(\Omega) = C_{calib}^2 S_{xx}(\Omega)$. This calibration factor is estimated using the equipartition theorem applied to the kinetic energy, E_{kin}, of the nanoparticle [14]

$$\langle E_{kin} \rangle = \frac{1}{2} m \frac{\langle \dot{V}^2 \rangle}{C_{calib}^2} = \frac{1}{2} k_B T \tag{8}$$

where the position variance is given by

$$\langle x^2 \rangle = \int_0^\infty d\Omega S_{xx}(\Omega) \tag{9}$$

C_{calib} is computed for each wavefront along the three axes, assuming a $T = 300$K temperature at high pressures (> 1 mbar). Temporal traces are filtered around the resonance frequency of each axis, yielding the particle's position relative to its equilibrium position. Velocities and accelerations are then calculated using a Gaussian kernel function, which minimizes noise by applying a locally weighted average to the data. This calibration method ensures accurate conversion of raw sensor data into real physical quantities, enabling reliable measurements of velocities and accelerations even in the presence of experimental noise.

4.3 Experimental results

Figure S16**a** and **b** illustrate, for respectively a uniform and an optimized wavefront (see insets), the probability currents in the position space (ρ, z), where $\rho = \sqrt{x^2 + y^2}$ represents the transverse axis

in cylindrical coordinates. We clearly observe that the currents display vortices, whose amplitudes are altered when the optimized wavefront is applied. Figure S16**c** and **d** display, for respectively a uniform and optimized wavefront, the probability currents in the position space (x, y). These results demonstrate more pronounced confinements of the particle's distributions in all spatial directions when using the optimized wavefront (i.e., indicating enhanced trapping stiffnesses). Additionally, the amplitude of the Brownian vortices is significantly altered, particularly in the (ρ, z) space, reflecting the impact of wavefront optimization onto scattering forces.

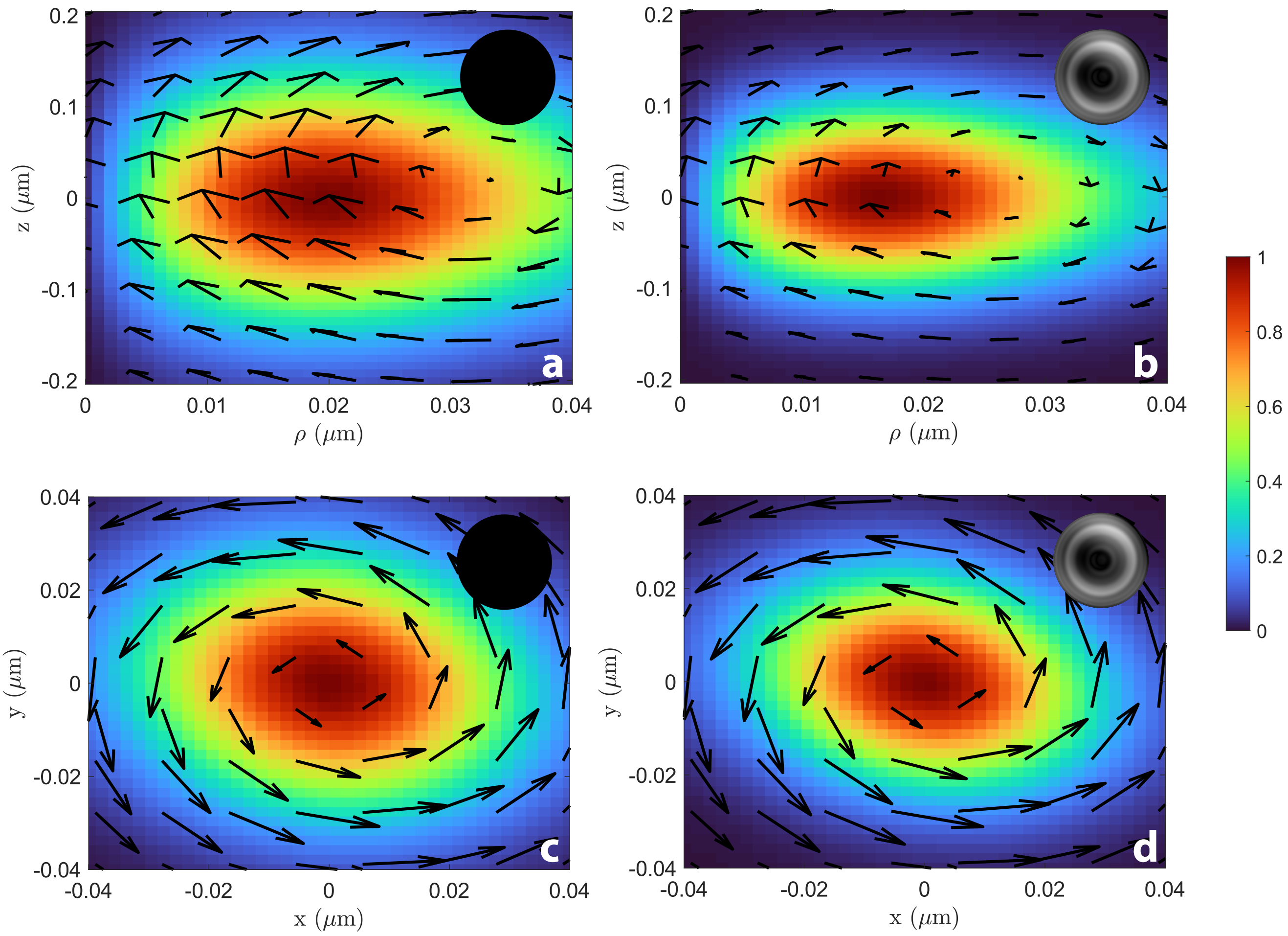

Figure S16: **Scattering force-induced vortices in position space**. Probability distributions P_x (colored map) and currents $\overline{J_x}$ (black arrows) in the space (ρ, z) for a 125 nm radius particle trapped at 1 mbar using a uniform (**a**) and an optimized wavefront (**b**). Probability distributions P_x (colored map) and currents $\overline{J_x}$ (black arrows) in the space (x, y) for a 125 nm radius particle trapped at 1 mbar using a uniform (**c**) and an optimized wavefront (**d**). The amplitudes of the distributions P_x are normalized to their maximum values.

When the pressure is reduced, Fig. S17 compares the vortex amplitudes measured in velocity space for the three phase patterns obtained with the optimizations displayed in Fig. S6. Although the amplitude increases consistently in the space (v_x, v_y) compared to the uniform wavefront, this trend is not observed in the space (v_ρ, v_z). These results suggest that wavefront shaping can be leveraged to modulate optical scattering effects.

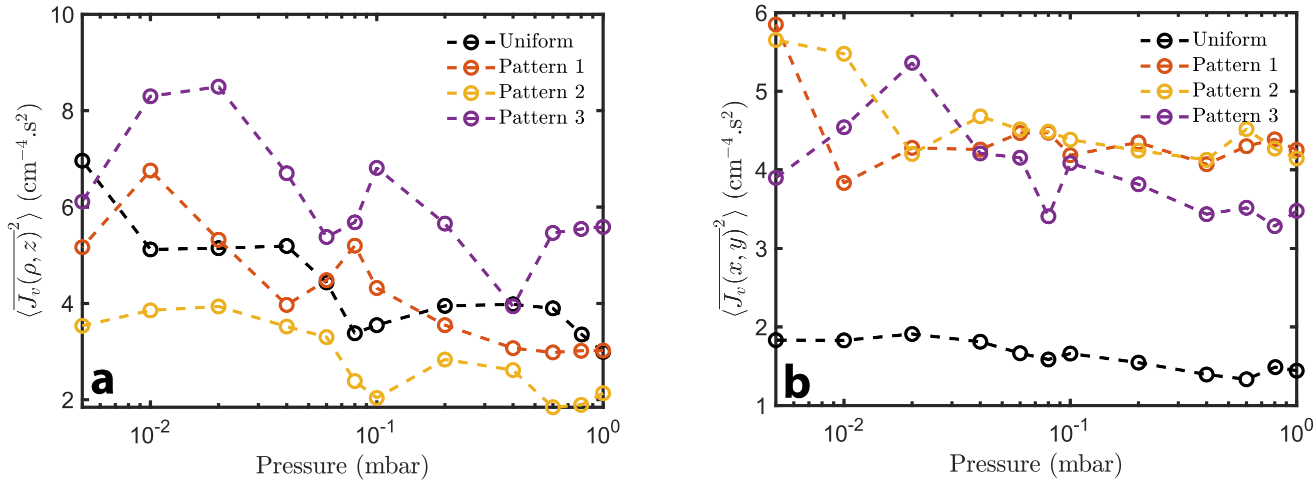

Figure S17: **Vortices in velocity space**. Amplitude of the vortices as a function of pressure for a 110 nm radius particle, using the wavefronts obtained from the 3 optimizations provided in Fig. S6. Panel **a** considers the space (v_ρ, v_z), while panel **b** considers the space (v_x, v_y).

5 Nonlinearities

5.1 Duffing broadening

When the pressure drops into the vacuum chamber, the nanoparticle experiences nonlinear oscillations that originate from non-quadratic components of the optical confinement[15]. This translates into Duffing nonlinearities and the equation of motion of a levitated nanoparticle of mass m along a direction $q_i \in [x, y, z]$ reads

$$\ddot{q}_i + \Gamma_i \dot{q}_i + \Omega_i^2 q_i \left(1 - \sum_{j\in[x,y,z]} \xi_j q_j^2\right) = \frac{F_{fl}(t)}{m} \tag{10}$$

in which Ω_i, Γ_i and ξ_j stand for the mechanical frequency, the damping of the surrounding gas and the Duffing nonlinearities, respectively. In equation (10), F_{fl} stands for a Langevin force related to Γ_i through the fluctuation-dissipation theorem. The damping provided by the surrounding gas is proportional to its pressure P and fulfills

$$\Gamma_i = \frac{64 r_i^2}{3m\bar{v}} P \tag{11}$$

where r_i and $\bar{v}$ correspond to the nanoparticle's dimension along q_i and the averaged velocity of the gas' molecules, respectively. Below, we drop the subscript "i" for simplicity.

The evolution of the spectral linewidth as a function of pressure (Figure 4 of the main text) is governed by two competing mechanisms, which we will respectively refer to as linear, $\Delta\Omega_L$, and nonlinear broadening, $\Delta\Omega_{NL}$. Set by gas damping, the linear broadening reads $\Delta\Omega_L = \Gamma$ and is therefore proportional to pressure. Resulting from Duffing nonlinearities, the nonlinear broadening reads $\Delta\Omega_{NL} = 3/4 k_B T\xi/\Omega$, in which T stands for the internal temperature of the particle. At high pressures, $\Delta\Omega_L$ dominates over $\Delta\Omega_{NL}$. At low pressures, $\Delta\Omega_L$ becomes smaller than $\Delta\Omega_{NL}$, which then becomes the dominant broadening mechanism.

5.2 Nonlinearity reduction at low pressure

To characterize the nonlinearities displayed in Figure 4 of the main text, we analyze the frequency fluctuations as a function of pressure. Following the approach of [15], the resonance frequency is extracted from short-time PSDs computed over 10 ms intervals. The statistical distribution of resonance

frequencies was then determined from a 20 s total trace, yielding a dataset of 2000 traces per pressure value. Figure S18**a** shows histograms of resonance frequencies for a uniform (black) and an optimized (red) wavefront along each axis at 0.1 mbar (using the same nanoparticle studied in the main article). In Figure 4**c** of the main text, we plot the standard deviation σ_f of these distributions as a function of pressure. As a consistency check regarding the reduction of nonlinearities, we reproduce in Fig. S18**b** the same approach using a different 125 nm particle.

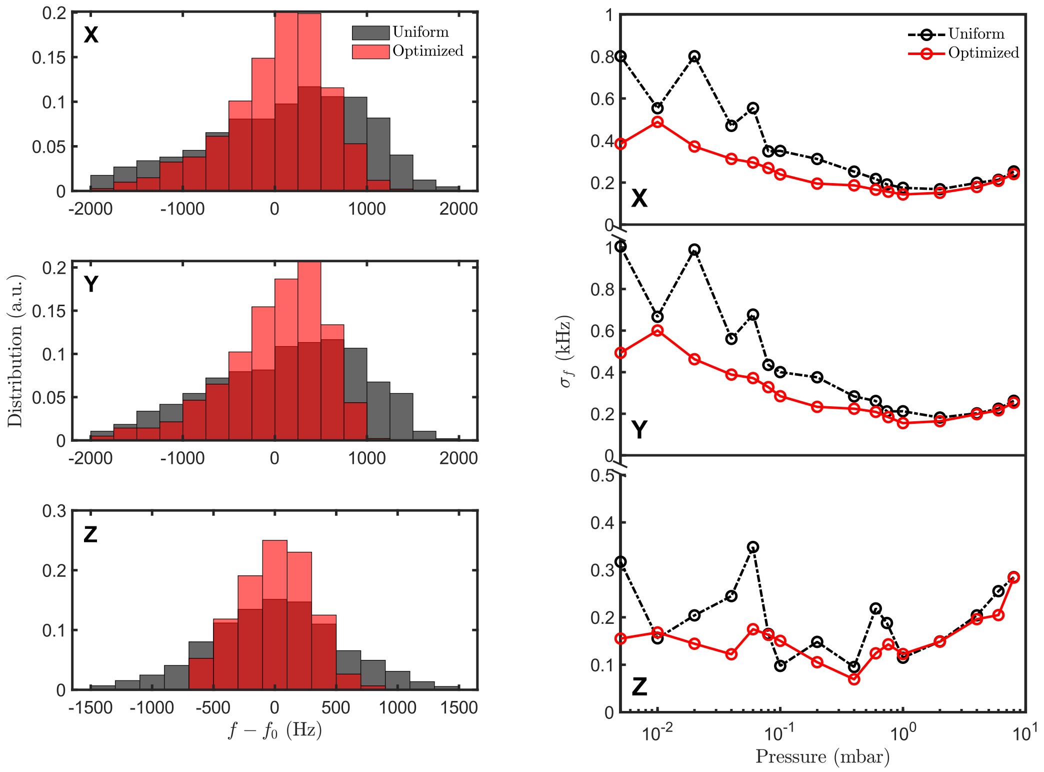

Figure S18: **Nonlinearities.** **a** Histograms of the resonance frequency distribution at 0.1 mbar for the three axes, comparing a uniform (black) and an optimized wavefront (red). **b** Standard deviations, σ_f, of 10 ms time-trace frequency distributions measured along x, y and z at different pressures for a uniform (black) and an optimized (red) wavefront.

Figure 4**c** of the main text as well as Fig. S18**b** show that, at high pressure (> 1 mbar), the uniform and optimized profiles enforce similar evolutions, while at lower pressure (< 1 mbar) spectral broadening is clearly reduced in the optimized case. As explained in section 5.1, at high pressure, spectral broadening (i.e., σ_f) identifies with $\Delta\Omega_L$ and is fully governed by the surrounding gas. Therefore, both uniform and optimized profile experience the same linear broadening. At lower pressure, broadening becomes nonlinear and evolves as $\Delta\Omega_{NL} \propto \xi/\Omega$. On top of enhancing the mechanical resonance, Ω, numerical simulations show (e.g., see Fig. S14) that the optimization makes the "effective" potential more quadratic and thus reduces the nonlinearities, ξ. As a result, $\Delta\Omega_{NL}$ is strongly reduced by the optimization, which leads to a weaker nonlinear broadening that emerges at a lower pressure. This demonstrates that wavefront shaping not only enhances trap stiffness but also provides a means to control and mitigate nonlinear effects.

In the model provided in section 5.1, below ≈ 1 mbar, nonlinear broadening should remain constant when the pressure is reduced. Nonetheless, we observe experimentally that this is not the case (e.g., see Fig. S18**b**). Already reported in [15], this evolution can have different origins, like the amplification of nonlinearities or the increase of the nanoparticle's internal temperature[16].

5.3 Preservation of stiffness enhancement at low pressure

At last, we confirm experimentally that the optimization remains effective at low pressure. When pressure is reduced, the resonance frequency along each axis tends to slightly drop. This decrease originates from Duffing nonlinearities, which increase when gas damping is reduced. This behavior can be observed in Fig. S19, where we plot the evolution of the resonances along all three axes when pressure is varied. We apply both the uniform (dashed black) and optimized (red) profile used in Figure 4 of the main text. In particular, one can see that the longitudinal direction z (along which optical confinement is weaker and therefore nonlinearities less pronounced) is barely affected by the drop in pressure. In contrast, the transverse directions x and y (more subject to nonlinearities) fluctuate slightly more. Yet, despite these small changes, the stiffness enhancements are not affected by the surrounding pressure.

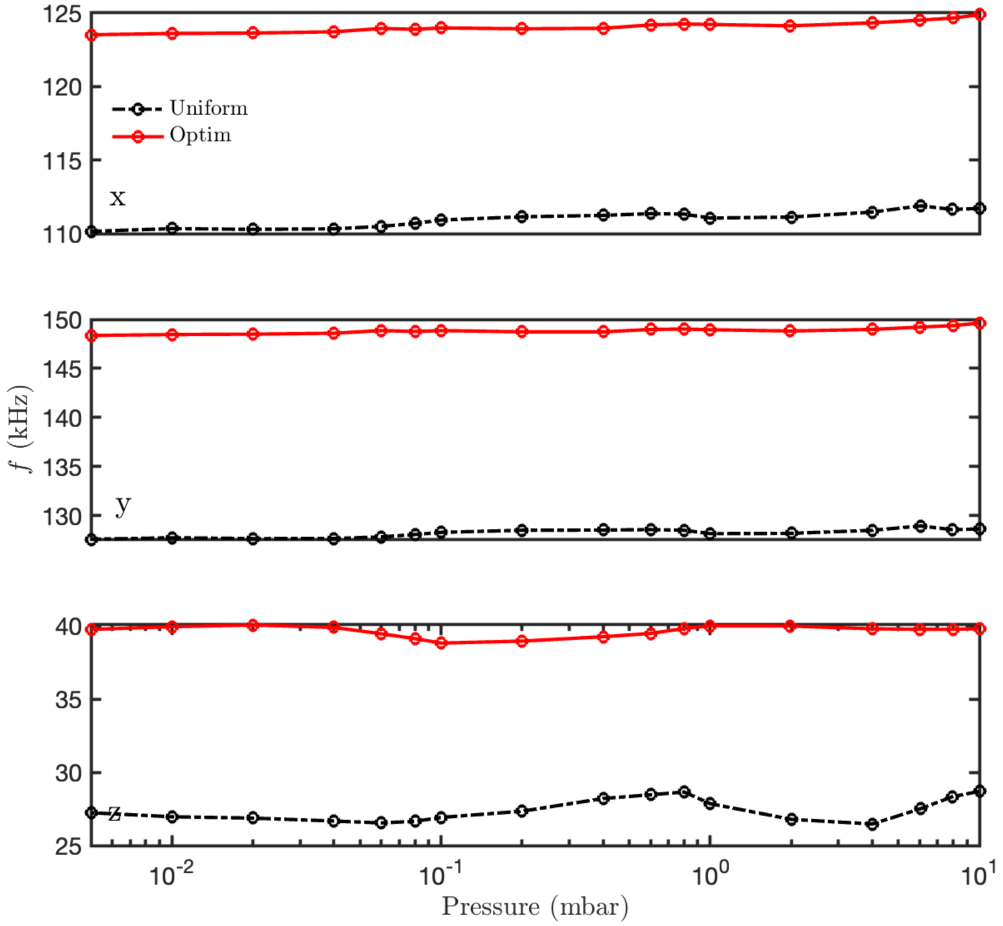

Figure S19: **Frequencies as a function of pressure**. When pressure is varied in the vacuum chamber, the black (respectively red) curves display the evolution of the resonances along x (top), y (middle) and z (bottom) under the uniform (respectively optimized) beam profile used in Figure 4 of the main text.

References

[1] Cuihong Li, Yuanyuan Ma, Jinchuan Wang, Qianwen Ying, Shaochong Zhu, Zhenhai Fu, Xinbing Jiang, Huan Yang, Tao Liang, Xiaowen Gao, and Huizhu Hu. Morphological tracking and tuning of silica nanoparticles in optomechanical systems for enhanced stable levitation in vacuum. *ACS Applied Nano Materials*, 7(22):25493–25499, 2024.

[2] Mingjie Zheng, Shengnan Chen, Bin Liu, Zuquan Weng, and Zhifang Li. Fast measurement of the phase flicker of a digitally addressable lcos-slm. *Optik*, 242, 9 2021.

[3] Kuan Hsu Fan-Chiang, Shang Hao Huang, Che Yung Shen, Hsing Lung Wang, Yuet Wing Li, Hsien Chang Tsai, and Yi Pai Huang. Analog lcos slm devices for ar display applications. *Journal of the Society for Information Display*, 28:581–590, 7 2020.

[4] Lukas Novotny and Bert Hecht. *Principles of nano-optics*. Cambridge university press, 2012.

[5] Gérard Gouesbet. Generalized lorenz–mie theories and mechanical effects of laser light, on the occasion of arthur ashkin's receipt of the 2018 nobel prize in physics for his pioneering work in optical levitation and manipulation: A review. *Journal of Quantitative Spectroscopy and Radiative Transfer*, 225:258–277, 2019.

[6] Marco Riccardi, Andrei Kiselev, Karim Achouri, and Olivier J.F. Martin. Multipolar expansions for scattering and optical force calculations beyond the long wavelength approximation. *Physical Review B*, 106, 9 2022.

[7] Jun Chen, Jack Ng, Zhifang Lin, and C. T. Chan. Optical pulling force. *Nature Photonics*, 5:531–534, 9 2011.

[8] Gérard Gouesbet, V.S. De Angelis, and Leonardo André Ambrosio. Optical forces and optical force categorizations on small magnetodielectric particles in the framework of generalized lorenz-mie theory. *Journal of Quantitative Spectroscopy and Radiative Transfer*, 279:108046, 2022.

[9] Fernando Monteiro, Sumita Ghosh, Adam Getzels Fine, and David C. Moore. Optical levitation of 10-ng spheres with nano-g acceleration sensitivity. *Physical Review A*, 96:063841, 12 2017.

[10] Peilong Hong and Willem L Vos. Controlled light scattering of a single nanoparticle by wave-front shaping. *Physical Review A*, 106(6):063502, 2022.

[11] Bo Sun, Jiayi Lin, Ellis Darby, Alexander Y Grosberg, and David G Grier. Brownian vortexes. *Physical Review E—Statistical, Nonlinear, and Soft Matter Physics*, 80(1):010401, 2009.

[12] Matthieu Mangeat, Yacine Amarouchene, Yann Louyer, Thomas Guérin, and David S. Dean. Role of nonconservative scattering forces and damping on brownian particles in optical traps. *Phys. Rev. E*, 99:052107, May 2019.

[13] Yacine Amarouchene, Matthieu Mangeat, Benjamin Vidal Montes, Lukas Ondic, Thomas Guérin, David S. Dean, and Yann Louyer. Nonequilibrium dynamics induced by scattering forces for optically trapped nanoparticles in strongly inertial regimes. *Phys. Rev. Lett.*, 122:183901, May 2019.

[14] Erik Hebestreit, Martin Frimmer, René Reimann, Christoph Dellago, Francesco Ricci, and Lukas Novotny. Calibration and energy measurement of optically levitated nanoparticle sensors. *Review of Scientific Instruments*, 89(3):033111, 03 2018.

[15] Jan Gieseler, Lukas Novotny, and Romain Quidant. Thermal nonlinearities in a nanomechanical oscillator. *Nature physics*, 9(12):806–810, 2013.

[16] Yacine Amarouchene, Matthieu Mangeat, Benjamin Vidal Montes, Lukas Ondic, Thomas Guérin, David S. Dean, and Yann Louyer. Nonequilibrium dynamics induced by scattering forces for optically trapped nanoparticles in strongly inertial regimes. *Physical Review Letters*, 122:1–6, 2019.
